# 3-[(3,4-Dichloro­phen­yl)amino­carbon­yl]propionic acid

**DOI:** 10.1107/S1600536809015025

**Published:** 2009-04-25

**Authors:** Farooq Ali Shah, M. Nawaz Tahir, Saqib Ali, Sajjad Ahmed, Muhammad Danish

**Affiliations:** a Department of Chemistry, Quaid-i-Azam University, Islamabad 45320, Pakistan; b Department of Physics, University of Sargodha, Sargodha, Pakistan; c Department of Chemistry, University of Sargodha, Sargodha, Pakistan

## Abstract

In the title compound, C_10_H_9_Cl_2_NO_3_, inversion dimers occur due to pairs of inter­molecular O—H⋯O hydrogen bonds from the carboxyl groups forming *R*_2_^2^(8) loops. The dimers are linked into *C*(4) chains along the *a* axis by inter­molecular N—H⋯O links. A short intra­molecular C—H⋯O contact occurs in the mol­ecule.

## Related literature

For a related structure, see: Shah *et al.* (2008 ▶). For background, see: Pellerito & Nagy (2002 ▶). For graph-set notation, see: Bernstein *et al.* (1995 ▶).
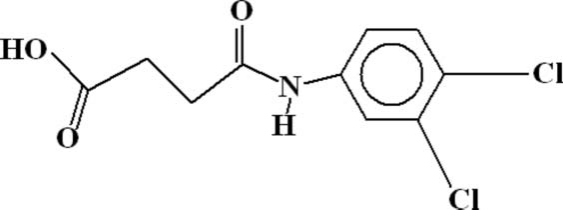


## Experimental

### 

#### Crystal data

C_10_H_9_Cl_2_NO_3_
*M* *_r_* = 262.08
Monoclinic, 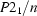

*a* = 4.8441 (4) Å
*b* = 10.3388 (10) Å
*c* = 22.457 (2) Å
β = 90.613 (3)°
*V* = 1124.62 (17) Å^3^
*Z* = 4
Mo *K*α radiation
μ = 0.57 mm^−1^
*T* = 296 K
0.25 × 0.12 × 0.10 mm

#### Data collection

Bruker Kappa APEXII CCD diffractometer
Absorption correction: multi-scan (*SADABS*; Bruker, 2005 ▶) *T*_min_ = 0.925, *T*_max_ = 0.949
11915 measured reflections
2912 independent reflections
2028 reflections with *I* > 3σ(*I*)
*R*_int_ = 0.028

#### Refinement

*R*[*F* ^2^ > 2σ(*F* ^2^)] = 0.065
*wR*(*F* ^2^) = 0.182
*S* = 1.05
2912 reflections
172 parameters
Only H-atom coordinates refined
Δρ_max_ = 0.89 e Å^−3^
Δρ_min_ = −0.84 e Å^−3^

### 

Data collection: *APEX2* (Bruker, 2007 ▶); cell refinement: *SAINT* (Bruker, 2007 ▶); data reduction: *SAINT*; program(s) used to solve structure: *SHELXS97* (Sheldrick, 2008 ▶); program(s) used to refine structure: *SHELXL97* (Sheldrick, 2008 ▶); molecular graphics: *ORTEP-3* (Farrugia, 1997 ▶) and *PLATON* (Spek, 2009 ▶); software used to prepare material for publication: *WinGX* (Farrugia, 1999 ▶) and *PLATON*.

## Supplementary Material

Crystal structure: contains datablocks global, I. DOI: 10.1107/S1600536809015025/hb2958sup1.cif

Structure factors: contains datablocks I. DOI: 10.1107/S1600536809015025/hb2958Isup2.hkl

Additional supplementary materials:  crystallographic information; 3D view; checkCIF report

## Figures and Tables

**Table 1:** Hydrogen-bond geometry (Å, °)

*D*—H⋯*A*	*D*—H	H⋯*A*	*D*⋯*A*	*D*—H⋯*A*
N1—H1*N*⋯O3^i^	0.81 (3)	2.10 (3)	2.887 (3)	165 (3)
O1—H1*O*⋯O2^ii^	0.80 (4)	1.87 (4)	2.665 (3)	170 (4)
C6—H6⋯O3	0.88 (4)	2.58 (4)	2.960 (4)	107 (3)

Symmetry codes: (i) 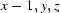; (ii) 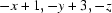.

**Fig. 1.**
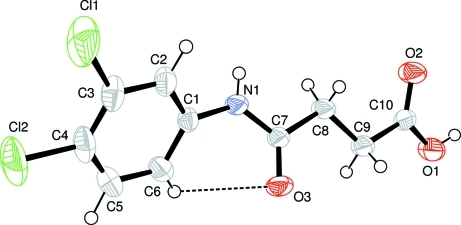
View of (I) with displacement ellipsoids drawn at the 50% probability level. H-atoms are shown by small spheres of arbitrary radius. The dashed lines represent the intramolecular H-bonds.

**Fig. 2.**
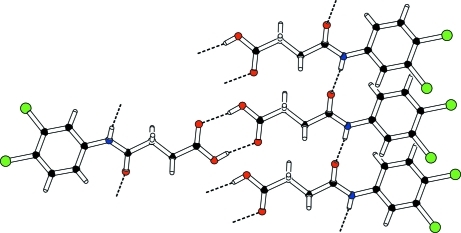
The partial unit cell packing of (I) showing the dimeric nature and the linkage of dimers along the a axis.

## supplementary crystallographic information

### Comment

In order to get a better insight in how the metallic species behave inside the biological systems, it is necessary to study their coordination behavior with biomolecules *i.e.* ligands having hetero-donor oxygen and nitrogen atoms (Pellerito & Nagy, 2002). Therefore, the title compound (I) has been prepared for the study of complexation with different metals.

The title compound is the structural isomer of 3-(3,5-dichloroanilinocarbonyl) propionic acid (Shah *et al.*, 2008). Due to the change of chloro substitution, the packing of the title compound has been changed. In this structure there does not exist any kind of π-interaction. The dimeric nature and the linkage of the dimers in title compound is in agreement with the reported structural isomer. In (I) the C==O bond distances for carboxylate and carbonyl group have values of (C10==O2: 1.236 (3) Å) and (C7==O3: 1.214 (3) Å), and in comparison to 1.219 (3) and 1.225 (2) Å, respectively. The C—N bond distances are compareable within experimental errors. In both compounds similar intermolecular H-bonding (Table 2, Fig. 2) has been observed. The dihedral angle between the aromatic ring (C1—C6) and (C8—C10/O1/O2) have a value of 20.45 (20)°, whereas with (C1/N1/C7/O3) its value is 39.02 (16)°. The value of dihedral angle between (C8—C10/O1/O2) and (C1/N1/C7/O3) is 18.69 (18)°. There exist an intramolecular H-bond of C—H···O type and completes a six-membered heterocyclic ring adjacent to the benzene ring. There does not exist any kind of π-interactions.

### Experimental

3,4-dichloroanilline (16.2 g, 0.1 mol) and succinic anhydride (10 g, 0.1 mole) were mixed in glacial acetic acid and stirred overnight. The solution was filtered and precipitated material was washed with distilled water. The acid formed was recrystallized from acetone to yield colourless blocks of (I). (Yield: 80%).

### Crystal data

**Table:**

C_10_H_9_Cl_2_NO_3_	*F*(000) = 536
*M**_r_* = 262.08	*D*_x_ = 1.548 Mg m^−^^3^
Monoclinic, *P*2_1_/*n*	Mo *K*α radiation, λ = 0.71073 Å
Hall symbol: -P 2yn	Cell parameters from 2912 reflections
*a* = 4.8441 (4) Å	θ = 2.7–28.9°
*b* = 10.3388 (10) Å	µ = 0.57 mm^−^^1^
*c* = 22.457 (2) Å	*T* = 296 K
β = 90.613 (3)°	Block, colourless
*V* = 1124.62 (17) Å^3^	0.25 × 0.12 × 0.10 mm
*Z* = 4	

### Data collection

**Table:**

Bruker Kappa APEXII CCD diffractometer	2912 independent reflections
Radiation source: fine-focus sealed tube	2028 reflections with *I* > 3σ(*I*)
graphite	*R*_int_ = 0.028
Detector resolution: 7.5 pixels mm^-1^	θ_max_ = 28.9°, θ_min_ = 2.7°
ω scans	*h* = −6→6
Absorption correction: multi-scan (*SADABS*; Bruker, 2005)	*k* = −13→13
*T*_min_ = 0.925, *T*_max_ = 0.949	*l* = −30→30
11915 measured reflections	

### Refinement

**Table:**

Refinement on *F*^2^	Primary atom site location: structure-invariant direct methods
Least-squares matrix: full	Secondary atom site location: difference Fourier map
*R*[*F*^2^ > 2σ(*F*^2^)] = 0.065	Hydrogen site location: inferred from neighbouring sites
*wR*(*F*^2^) = 0.182	Only H-atom coordinates refined
*S* = 1.05	*w* = 1/[σ^2^(*F*_o_^2^) + (0.0667*P*)^2^ + 1.6338*P*] where *P* = (*F*_o_^2^ + 2*F*_c_^2^)/3
2912 reflections	(Δ/σ)_max_ < 0.001
172 parameters	Δρ_max_ = 0.89 e Å^−^^3^
0 restraints	Δρ_min_ = −0.84 e Å^−^^3^

### Special details

**Table:**

Geometry. Bond distances, angles *etc*. have been calculated using the rounded fractional coordinates. All su's are estimated from the variances of the (full) variance-covariance matrix. The cell e.s.d.'s are taken into account in the estimation of distances, angles and torsion angles
Refinement. Refinement of *F*^2^ against ALL reflections. The weighted *R*-factor *wR* and goodness of fit *S* are based on *F*^2^, conventional *R*-factors *R* are based on *F*, with *F* set to zero for negative *F*^2^. The threshold expression of *F*^2^ > σ(*F*^2^) is used only for calculating *R*-factors(gt) *etc*. and is not relevant to the choice of reflections for refinement. *R*-factors based on *F*^2^ are statistically about twice as large as those based on *F*, and *R*- factors based on ALL data will be even larger.

### Fractional atomic coordinates and isotropic or equivalent isotropic displacement parameters (Å^2^)

**Table:**

	*x*	*y*	*z*	*U*_iso_*/*U*_eq_	
Cl1	0.2707 (3)	0.56808 (11)	0.26822 (5)	0.0954 (5)	
Cl2	0.6942 (3)	0.37607 (10)	0.20648 (6)	0.0945 (5)	
O1	0.7844 (5)	1.3991 (2)	−0.01026 (13)	0.0584 (9)	
O2	0.4038 (4)	1.3685 (2)	0.04204 (12)	0.0551 (8)	
O3	0.9245 (4)	0.9675 (2)	0.08819 (12)	0.0546 (8)	
N1	0.4948 (5)	0.9009 (2)	0.11088 (11)	0.0372 (7)	
C1	0.5572 (5)	0.7772 (3)	0.13420 (12)	0.0341 (8)	
C2	0.4079 (7)	0.7353 (3)	0.18269 (14)	0.0427 (9)	
C3	0.4538 (8)	0.6133 (3)	0.20585 (14)	0.0500 (10)	
C4	0.6432 (8)	0.5320 (3)	0.18016 (16)	0.0543 (11)	
C5	0.7934 (8)	0.5742 (3)	0.13222 (18)	0.0568 (11)	
C6	0.7517 (7)	0.6968 (3)	0.10907 (15)	0.0448 (9)	
C7	0.6785 (5)	0.9883 (3)	0.09118 (12)	0.0345 (8)	
C8	0.5505 (6)	1.1164 (3)	0.07282 (17)	0.0438 (9)	
C9	0.7506 (6)	1.2040 (3)	0.04237 (17)	0.0437 (9)	
C10	0.6312 (5)	1.3316 (3)	0.02430 (13)	0.0384 (8)	
H1N	0.334 (7)	0.922 (3)	0.1111 (15)	0.0447*	
H1O	0.722 (9)	1.470 (4)	−0.0157 (19)	0.0701*	
H2	0.278 (7)	0.793 (4)	0.2003 (15)	0.0513*	
H5	0.939 (8)	0.518 (4)	0.1166 (17)	0.0680*	
H6	0.846 (7)	0.722 (4)	0.0777 (16)	0.0537*	
H8A	0.489 (8)	1.153 (4)	0.1063 (16)	0.0527*	
H8B	0.377 (8)	1.101 (3)	0.0478 (15)	0.0527*	
H9A	0.899 (7)	1.225 (4)	0.0680 (16)	0.0523*	
H9B	0.840 (7)	1.164 (4)	0.0077 (16)	0.0523*	

### Atomic displacement parameters (Å^2^)

**Table:**

	*U*^11^	*U*^22^	*U*^33^	*U*^12^	*U*^13^	*U*^23^
Cl1	0.1600 (13)	0.0566 (6)	0.0704 (7)	−0.0127 (7)	0.0418 (7)	0.0207 (5)
Cl2	0.1284 (11)	0.0363 (5)	0.1187 (10)	0.0107 (6)	−0.0093 (8)	0.0277 (5)
O1	0.0476 (13)	0.0356 (12)	0.0924 (19)	0.0084 (10)	0.0234 (12)	0.0257 (12)
O2	0.0400 (11)	0.0410 (12)	0.0846 (17)	0.0114 (9)	0.0176 (11)	0.0226 (11)
O3	0.0251 (10)	0.0418 (12)	0.0969 (18)	0.0059 (8)	0.0058 (10)	0.0219 (12)
N1	0.0260 (10)	0.0317 (12)	0.0541 (14)	0.0044 (9)	0.0040 (10)	0.0114 (10)
C1	0.0337 (13)	0.0263 (12)	0.0423 (14)	−0.0008 (10)	−0.0030 (10)	0.0043 (10)
C2	0.0540 (17)	0.0294 (13)	0.0449 (16)	−0.0031 (12)	0.0066 (13)	0.0006 (12)
C3	0.073 (2)	0.0331 (15)	0.0440 (16)	−0.0104 (14)	0.0021 (15)	0.0056 (12)
C4	0.072 (2)	0.0276 (14)	0.063 (2)	0.0001 (14)	−0.0119 (17)	0.0098 (14)
C5	0.059 (2)	0.0345 (16)	0.077 (2)	0.0123 (15)	0.0027 (18)	0.0006 (16)
C6	0.0445 (16)	0.0358 (15)	0.0542 (18)	0.0066 (12)	0.0086 (13)	0.0038 (13)
C7	0.0279 (12)	0.0302 (13)	0.0455 (14)	0.0028 (10)	0.0029 (10)	0.0078 (11)
C8	0.0307 (14)	0.0320 (14)	0.069 (2)	0.0075 (11)	0.0112 (13)	0.0165 (14)
C9	0.0319 (14)	0.0315 (14)	0.068 (2)	0.0042 (11)	0.0091 (13)	0.0137 (13)
C10	0.0305 (13)	0.0305 (13)	0.0543 (16)	0.0010 (10)	0.0021 (11)	0.0090 (12)

### Geometric parameters (Å, °)

**Table:**

Cl1—C3	1.730 (4)	C4—C5	1.377 (5)
Cl2—C4	1.734 (3)	C5—C6	1.384 (5)
O1—C10	1.286 (4)	C7—C8	1.518 (4)
O2—C10	1.236 (3)	C8—C9	1.497 (5)
O3—C7	1.214 (3)	C9—C10	1.495 (4)
O1—H1O	0.80 (4)	C2—H2	0.96 (4)
N1—C1	1.414 (4)	C5—H5	0.98 (4)
N1—C7	1.346 (4)	C6—H6	0.88 (4)
N1—H1N	0.81 (3)	C8—H8A	0.90 (4)
C1—C6	1.382 (4)	C8—H8B	1.02 (4)
C1—C2	1.383 (4)	C9—H9A	0.94 (4)
C2—C3	1.382 (4)	C9—H9B	0.99 (4)
C3—C4	1.376 (5)		
			
C10—O1—H1O	112 (3)	C8—C9—C10	114.0 (2)
C1—N1—C7	126.1 (2)	O1—C10—O2	123.3 (3)
C7—N1—H1N	117 (2)	O1—C10—C9	114.8 (2)
C1—N1—H1N	116 (2)	O2—C10—C9	121.9 (3)
N1—C1—C6	122.5 (3)	C1—C2—H2	119 (2)
N1—C1—C2	117.6 (3)	C3—C2—H2	121 (2)
C2—C1—C6	119.9 (3)	C4—C5—H5	119 (2)
C1—C2—C3	119.9 (3)	C6—C5—H5	121 (2)
Cl1—C3—C2	118.0 (3)	C1—C6—H6	121 (3)
C2—C3—C4	120.4 (3)	C5—C6—H6	120 (3)
Cl1—C3—C4	121.6 (3)	C7—C8—H8A	106 (3)
C3—C4—C5	119.7 (3)	C7—C8—H8B	110.2 (18)
Cl2—C4—C5	119.1 (3)	C9—C8—H8A	111 (3)
Cl2—C4—C3	121.2 (3)	C9—C8—H8B	112.2 (19)
C4—C5—C6	120.5 (3)	H8A—C8—H8B	104 (3)
C1—C6—C5	119.7 (3)	C8—C9—H9A	111 (2)
O3—C7—N1	123.5 (3)	C8—C9—H9B	114 (2)
O3—C7—C8	122.6 (3)	C10—C9—H9A	105 (2)
N1—C7—C8	113.9 (2)	C10—C9—H9B	109 (2)
C7—C8—C9	112.8 (2)	H9A—C9—H9B	104 (3)
			
C7—N1—C1—C2	140.0 (3)	Cl1—C3—C4—C5	−177.0 (3)
C7—N1—C1—C6	−42.4 (4)	C2—C3—C4—Cl2	−177.3 (3)
C1—N1—C7—O3	4.2 (5)	C2—C3—C4—C5	2.0 (5)
C1—N1—C7—C8	−175.8 (3)	Cl2—C4—C5—C6	178.1 (3)
N1—C1—C2—C3	177.7 (3)	C3—C4—C5—C6	−1.2 (6)
C6—C1—C2—C3	0.0 (5)	C4—C5—C6—C1	−0.2 (5)
N1—C1—C6—C5	−176.8 (3)	O3—C7—C8—C9	9.0 (5)
C2—C1—C6—C5	0.8 (5)	N1—C7—C8—C9	−171.0 (3)
C1—C2—C3—Cl1	177.6 (2)	C7—C8—C9—C10	−179.4 (3)
C1—C2—C3—C4	−1.4 (5)	C8—C9—C10—O1	−169.1 (3)
Cl1—C3—C4—Cl2	3.7 (5)	C8—C9—C10—O2	11.6 (5)

### Hydrogen-bond geometry (Å, °)

**Table:**

*D*—H···*A*	*D*—H	H···*A*	*D*···*A*	*D*—H···*A*
N1—H1N···O3^i^	0.81 (3)	2.10 (3)	2.887 (3)	165 (3)
O1—H1O···O2^ii^	0.80 (4)	1.87 (4)	2.665 (3)	170 (4)
C6—H6···O3	0.88 (4)	2.58 (4)	2.960 (4)	107 (3)

Symmetry codes: (i) *x*−1, *y*, *z*; (ii) −*x*+1, −*y*+3, −*z*.
